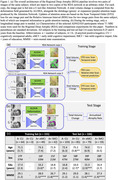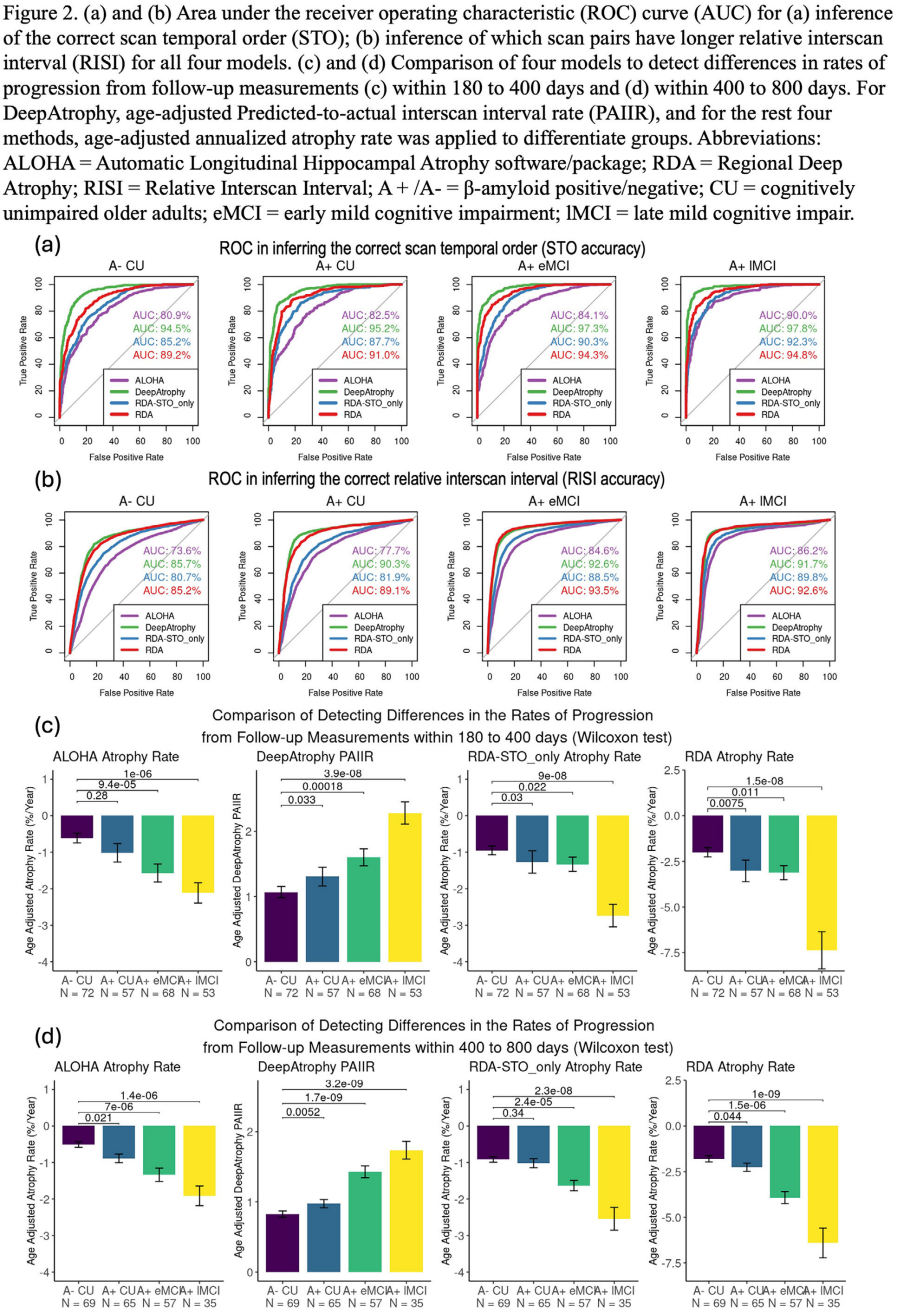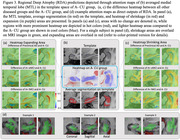# Regional Deep Atrophy: Using Temporal Information to Automatically Identify Regions Associated with Alzheimer’s Disease Progression from Longitudinal MRI

**DOI:** 10.1002/alz.095798

**Published:** 2025-01-09

**Authors:** Mengjin Dong, Long Xie, Laura Wisse, Robin de Flores, Sandhitsu R. Das, David A Wolk, Paul A. Yushkevich

**Affiliations:** ^1^ University of Pennsylvania, Philadelphia, PA USA; ^2^ Lund University, Lund Sweden; ^3^ Normandie Univ, UNICAEN, INSERM, U1237, PhIND “Physiopathology and Imaging of Neurological Disorders”, NeuroPresage Team, GIP Cyceron, Caen France

## Abstract

**Background:**

Assessment of longitudinal hippocampal atrophy is a well‐studied biomarker for Alzheimer’s disease (AD). However, most state‐of‐the‐art measurements calculate changes directly from MRI images using image registration/segmentation, which may misreport head motion or MRI artifacts as neurodegeneration. We present a deep learning method Regional Deep Atrophy (RDA) that (1) estimates atrophy sensitive to progression by quantifying time‐associated changes in images, especially in preclinical AD stage (as in DeepAtrophy (Dong et al., 2021)), and (2) identifies regions where longitudinal changes significantly influence temporal inference.

**Method:**

RDA was trained on longitudinal T1‐weighted MRI from 155 ADNI participants and evaluated on 326 participants (Figure 1(c)). During training, two image pairs from the same participant are fed into two instances of the RDA network in arbitrary temporal order. Within each RDA network, a U‐Net is applied to one image pair of arbitrary order to predict attention regions informative of shrinkage/expansion. Attention regions are used to mask a deformation field computed by ALOHA (Das et al., 2012), and derive a total volume change measurement for attention areas. The attention regions are optimized by the Scan Temporal Order (STO) loss for one scan pair to evaluate if volume changes align with input image order, and the Relative Interscan Interval (RISI) metric to determine if larger volume changes correspond to longer interscan intervals for the whole RDA model (Figure 1). Only one longitudinal image pair is required for testing, directly generating the total volume change as atrophy measurement.

**Result:**

RDA achieves the similar ability to detect differences in atrophy between stages on the AD continuum as DeepAtrophy, especially in preclinical AD (Figure 2), while having additional explainability in the form of heatmaps that summarize expansion/shrinkage regions in the brain that contribute to the RDA change measurement (Figure 3). These heatmaps, derived in a fully data‐driven manner, largely recapitulate the areas of atrophy and expansion in the MTL reported by prior studies.

**Conclusion:**

RDA has similar prediction accuracy as DeepAtrophy, but its additional interpretability makes it more acceptable for use in clinical settings, and may lead to more sensitive biomarkers for disease monitoring and progression understanding in preclinical AD.